# Exploring the Most Visible German Websites on Melanoma Immunotherapy: A Web-Based Analysis

**DOI:** 10.2196/10676

**Published:** 2018-12-13

**Authors:** Julia Brütting, Theresa Steeb, Lydia Reinhardt, Carola Berking, Friedegund Meier

**Affiliations:** 1 Department of Dermatology Dresden University Hospital and Medical Faculty Carl Gustav Carus Technical University of Dresden Dresden Germany; 2 Department of Dermatology and Allergy University Hospital LMU Munich Munich Germany

**Keywords:** melanoma, immunotherapy, internet, patient education, quality, readability, websites, reliability, information

## Abstract

**Background:**

Patients diagnosed with melanoma frequently search the internet for treatment information, including novel and complex immunotherapy. However, health literacy is limited among half of the German population, and no assessment of websites on melanoma treatment has been performed so far.

**Objective:**

The aim of this study was to identify and assess the most visible websites in German language on melanoma immunotherapy.

**Methods:**

In accordance with the common Web-based information-seeking behavior of patients with cancer, the first 20 hits on Google, Yahoo, and Bing were searched for combinations of German synonyms for “melanoma” and “immunotherapy” in July 2017. Websites that met our predefined eligibility criteria were considered for assessment. Three reviewers independently assessed their quality by using the established DISCERN tool and by checking the presence of quality certification. Usability and reliability were evaluated by the LIDA tool and understandability by the Patient Education Materials Assessment Tool (PEMAT). The Flesch Reading Ease Score (FRES) was calculated to estimate the readability. The ALEXA and SISTRIX tools were used to investigate the websites’ popularity and visibility. The interrater agreement was determined by calculating Cronbach alpha. Subgroup differences were identified by t test, U test, or one-way analysis of variance.

**Results:**

Of 480 hits, 45 single websites from 30 domains were assessed. Only 2 website domains displayed a German quality certification. The average assessment scores, mean (SD), were as follows: DISCERN, 48 (7.6); LIDA (usability), 40 (2.0); LIDA (reliability), 10 (1.6); PEMAT, 69% (16%); and FRES, 17 (14), indicating mediocre quality, good usability, and understandability but low reliability and an even very low readability of the included individual websites. SISTRIX scores ranged from 0 to 6872 and ALEXA scores ranged from 17 to 192,675, indicating heterogeneity of the visibility and popularity of German website domains providing information on melanoma immunotherapy.

**Conclusions:**

Optimization of the most accessible German websites on melanoma immunotherapy is desirable. Especially, simplification of the readability of information and further adaption to reliability criteria are required to support the education of patients with melanoma and laypersons, and to enhance transparency.

## Introduction

Melanoma incidence has been increasing worldwide [1,2]. In Germany, it accounts for about 4% of all cancer types and is the fifth most common malignancy [3]. Approvals of new effective therapies in the last decade have substantially expanded the treatment spectrum, especially for patients with melanoma and metastatic disease. One of these treatment options is immunotherapy, the application of which has resulted in pioneering response rates and markedly increased survival chances of patients with melanoma [4-6].

Given the novelty, complexity, and potential toxicity of immunotherapy, the need for educating patients with melanoma has been increasing. Physicians serve as the primary information source in this context. However, recent research suggests that the information-seeking behavior of patients with melanoma [7-9] and the resources they use have been changing alongside with the application of modern media and structural changes in health care provision. Besides medical consultations and written information [10], as cancer patients’ persisting primary and most important sources of health information, a growing preference of the internet to acquire disease-specific information has been observed [8-11]. The search engines Google, Bing, and Yahoo are the most searched in this respect by the public and patients [12-15]. However, although the internet becomes increasingly popular, many patients with cancer are skeptical of Web-based information [16,17]. Not all websites on cancer issues are prepared by health professionals or medical and health care authorities, and the reliability and accuracy of the information available remain questionable [18]. Web-based information for patients with melanoma was previously found to be difficult to read [16], did not provide complete basic and transparent information, or contained misinformation [19].

It has become a common practice to review the most visible Web-based cancer information on therapy using scientifically validated tools [20-24] to explore what shortcomings exist and what should be considered when using the information. Web-based treatment information is used by patients to support their treatment decision making [25]. Therefore, genuine information should be presented in a complete and simple manner. The aim of this study was to explore first, what websites with information on melanoma immunotherapy in German language are currently visible and accessible at most when applying common Web-based search engines, and then to assess them in terms of their quality, reliability, usability, understandability, readability, visibility, and popularity. The results of this study will be beneficial for dermatologists to recommend and for patients with melanoma to identify appropriate websites with information on immunotherapy. Moreover, the results will indicate potential issues that providers should address to improve their websites.

## Methods

### Search Strategy

In accordance with common Web-based search patterns of the general population, including patients with cancer [12-15], 2 independent researchers (JB and TS) searched the first 20 hits on the most frequently used Web-based search engines Google, Bing, and Yahoo for a combination of German synonyms for “melanoma” and “immunotherapy.” *A priori*, Google trends analysis was used to identify relevant search combinations that people frequently used when searching Google for this topic. The search terms were adapted according to the Google trends analysis and were combined as “Melanom + Immuntherapie,” “Malignes Melanom + Immuntherapie,” “Hautkrebs + Immuntherapie,” and “Schwarzer Hautkrebs + Immuntherapie.” The search was performed between July 10 and July 14, 2017, using the Web-based browsers *Internet Explorer version 11* or *Mozilla Firefox version 57*.

### Inclusion and Exclusion Criteria

To be eligible for assessment, websites had to meet the following inclusion criteria: (1) contain information on immunotherapy referring to melanoma; (2) contain at least 5 sentences of information; (3) be accessible for free and for all users (including patients and laypersons); and (4) information is provided in German language. Websites were excluded if they were solely patient exchange platforms, advertising websites, conference or congress websites (eg, of medical conferences), websites dealing with nonmelanoma skin cancer (NMSC), websites about melanoma and NMSC in animals, websites solely providing videos or images, and websites of restricted access (eg, asking for log-in).

All hits of the search engine queries were screened for duplicates, and the predefined eligibility criteria were applied. Whenever discrepancies on the relevance of a website arose, a third researcher (FM or CB) was consulted as arbiter for resolution.

### Grouping of Websites

Owing to the variability in the creators of websites and for comparison, the websites were grouped by application of 2 different approaches, similar to Azer et al [20]. First, the websites were grouped according to their providers as follows: (1) commercial and pharmaceutical companies; (2) noncommercial or charity providers; (3) medical or scientific providers; (4) general public press; (5) commercial health information services; (6) clinics or health professionals; and (7) Wikipedia.org. The categorization was conducted independently by 2 researchers (JB and TS); disagreements were discussed and remedied in a subsequent meeting. Second, the groups (1)-(3) and (6) were summarized as oncology expert domains, and the groups (4), (5), and (7) were summarized as domains provided by the general public press. “General public press” domains describe domains that do not primarily address a particular subgroup of users, such as patients or medical experts, but the general public; these are usually provided by media such as news magazines, tabloids, and radio or television channels.

### Data Management and Website Assessment

The available baseline information (URL, title, name of the website provider, and year of publication) of each included website was documented. For inaccessible information, the tool Whois Lookup was used to complete the data collection.

Assessment was performed on the individual website level and the domain level, depending on the assessment tool.

Three reviewers (JB, TS, and LR) independently assessed the websites’ quality of information, usability, reliability, and understandability by applying different validated tools. Prior to the final assessment of the websites, the 3 reviewers piloted the use of the assessment tools by independently evaluating individual websites on NMSC to discuss potential difficulties or points of disagreement and resolve questions. The degree of agreement between all 3 reviewers for the final assessment was quantified by an interrater agreement analysis.

Furthermore, the readability of individual websites, as well as the visibility and popularity of the domains, was determined by using established calculating Web-based tools. The baseline information was extracted to an internally piloted data extraction sheet using Microsoft Excel 2010. The German melanoma guideline [26] was used as reference standard to check the scientific accuracy of a websites’ content.

### Quality of Information Assessment

The DISCERN tool (discern.de) is commonly used to assess the quality of information on cancer [20,24,27] and was developed for use by laypersons [28]. It consists of 16 items to review (1) a publication’s transparency (items 1-8); (2) content (items 9-15); and (3) to give an intuitive assessment summary (item 16). Items are scored on a 5-point scale ranging from 1 (“criterion is not met at all”) to 5 (“criterion is fully met”). An overall score of 80 and a summary mean score of 5, respectively, corresponds to the high quality of a publication (Table 1).

In addition, the presence of a health information quality certification, such as HONcode from the Health On the Net Foundation, Public Health Foundation (German: Stiftung Gesundheit) certificate, or the certificate from afgis (German: Aktionsforum Gesundheitsinformationssystem e.V.), was documented for each domain as well. Quality certifications on health topics are used to indicate that a domain meets particular quality criteria (eg, for transparency, reliability, and funding) and are usually awarded by charitable associations.

### Assessment of Usability and Reliability

LIDA is a validation tool for health care websites. It contains 41 items [29] for the assessment of the *accessibility*, *usability*, and *reliability* of domains on health topics. Each item can be rated with a score of 0 (“never”), 1 (“sometimes”), 2 (“mostly”), or 3 (“always”). We only assessed the domains’ *usability* (items 7-24 assessing the clarity of information, consistency of the domain design, the presence of effective browsing and search functions, and the presence of media) and *reliability* (items 25-41, assessing the domain update frequency, conflicts of interest, the methodology of the content production, and the accuracy of content). As we excluded websites with restricted access and also because of the unavailability of the basic corresponding LIDA category during our assessment, we decided to leave the category *accessibility* out. The overall LIDA score was calculated as a sum of the 2 mentioned categories, resulting in a maximum score of 81.

### Assessment of Understandability and Actionability

The Patient Education Materials Assessment Tool (PEMAT) [30,31] was used to assess the individual websites’ *understandability*. The *understandability* part comprises 17 items that cover content, word choice and style, numbers, structure, layout and design, and the use of visual aids. Another part covers *actionability* by 7 items. Each item can be scored as 0 (“disagree”), 1 (“agree”), or N/A (“not applicable”). Then, percentages of fulfilled items are calculated. The higher the value, the more understandability elements are applied on a website.

**Table 1:** Overview of assessment tools.

Category assessed		Tool used	Level of analysis	Score range	Interpretation of results
Quality		DISCERN	Individual websites	1-80	Higher values indicate higher quality
		Check for the Presence of Quality certificates	Domains	Presence or absence	
**Validity**		LIDA	Domains	0-81	Higher values indicate higher validity
	Usability			0-54	Higher values indicate better usability
	Reliability			0-27	Higher values indicate higher reliability
Understandability		PEMAT^a^	Individual websites	0%-100%	Higher percentage indicate higher understandability
Readability		FRES^b^	Individual websites	<0 to >60	Higher values indicate better readability <20 very low, 21-40 low, 41-60 average, >60 easy
Visibility		SISXTRIX	Domains	0-max	Higher values indicate higher visibility
Popularity		ALEXA	Domains	1-max	Lower values indicate better popularity rank

^a^PMAT: Patient Education Materials Assessment Tool.

^b^FRES: Flesch Reading Ease Score.

### Readability Analysis

Since the websites under investigation are accessible for laypersons and patients with melanoma, we evaluated whether they provide information in an appropriate readability level and whether they cover the general public and patients’ readability needs. Consistent with Azer et al [20], we analyzed a sample of 200-500 words or 4-5 sentences if the information was presented in up to 10 sentences. We then calculated the Flesch Reading Ease score (FRES) by using a Web-based tool adaptation for German texts [32] to determine the *readability* of individual websites. If the text was >10 sentences, we randomly extracted 4-5 connected sentences and 200-500 words, respectively. The score was calculated by using a formula that takes into account the word and sentence length, as well as characters and syllables per word, resulting in an absolute score that expresses the readability of a text ranging from <20 (very difficult), 21-30 (difficult), 31-40 (fairly difficult), 41-60 (standard), 61-70 (fairly easy), 71-80 (easy), and >80 (very easy). Owing to the FRES formula, it is possible that negative values may be results of the calculation, if, for example, sentence or words are very long:

FRES=206,835−84.6×average length of words (number of syllables)−1015×average length of sentences (number of words).

### Popularity and Visibility Analysis

In order to have a reference to a domain’s *popularity* and *visibility*, the ALEXA traffic tool [33] and the SISTRX tool [34] were used, respectively. We determined the domains’ ALEXA traffic rank in Germany, which is calculated through a combination of average daily visitors and pageviews on this domain over the past 3 months, that is, the domain with the highest combination is rated as number one. In addition, we estimated the daily pageviews per visitor and the time users spent on the domain. The SISTRIX visibility index is a measure of a domain’s discoverability within the search results in Google. The higher the value, the more visitors browse the domain.

### Statistical Analysis

Statistical analyses were conducted by using SPSS (IBM SPSS Statistics version 24, IBM Corporation, Armonk, NY, USA). Descriptive analyses included mean (SD) or median and interquartile ranges (IQR). Subgroup differences were explored by means of the *t* test or *U* test and by one-factor analysis of variance or the Kruskal–Wallis test. Statistical significance was set at *P* ≤.05. The interrater agreement of the 3 reviewers was determined using the intraclass correlation coefficient, as well as by determining the interitem correlations *r* between the individual reviewers.

## Results

### Identification of Eligible Websites

Our initial search in Google, Yahoo, and Bing identified 480 individual websites.

Using a multistep process, we screened the 480 websites for duplicates and checked them for accordance with our eligibility criteria. Multimedia Appendix 1 presents the detailed identification process of eligible websites. Finally, 45 individual websites provided by 30 domains met our eligibility criteria and were considered for the assessment.

### Grouping of Websites

Of 45 individual websites considered, 6 could be assigned to pharmaceutical companies, 4 to a noncommercial provider, 13 to medical science providers, 11 to the general public press, 7 to commercial health information services, 2 to Wikipedia, 1 to a hospital, and 1 to a health professional. Multimedia Appendix 2 lists all analyzed websites.

### Baseline Information of the Websites

The individual websites were published between 2007 and 2017 with 44% (20/45) published in 2017 and 56% (25/45) in the years before. The oldest websites were provided by Pharmazeutische Zeitung (2007) and the University Hospital of Ulm (2008; Multimedia Appendix 2; #11 and #26). Two-thirds (n=30) provided information on immunotherapy of melanoma and one-third (n=15) reported on immunotherapy in cancer, including melanoma.

### Quality of Information

#### Presence of Quality Certification

The domain krebsgesellschaft.de had a HONcode and an afgis quality certificate, and the domain apotheken-umschau.de displayed an afgis certificate, as well as a certificate of the Public Health Foundation [Stiftung Gesundheit] (Multimedia Appendix 2; #2 and #5). The other domains had no certificate.

#### DISCERN Results

Out of a total of 80 points, the 45 individual websites scored between 35 and 63 points. The mean DISCERN scores ranged from 2.1 to 3.7 points, indicating a medium–low to medium–high quality. Most score deductions were because of lacking information on nontreatment (item 12), on the potential impact of treatment on the patients’ quality of life (item 13) and the lack of information on scientifically uncertain aspects of treatment (item 8; Multimedia Appendix 3). The lowest DISCERN score was obtained from the website medecon.ruhr (35 points), and the highest score from wikipedia.org and uniklinik-ulm.de (63 points each; Multimedia Appendix 2; #22 and #8).

### Usability and Reliability—LIDA Results

**Figure 1:**
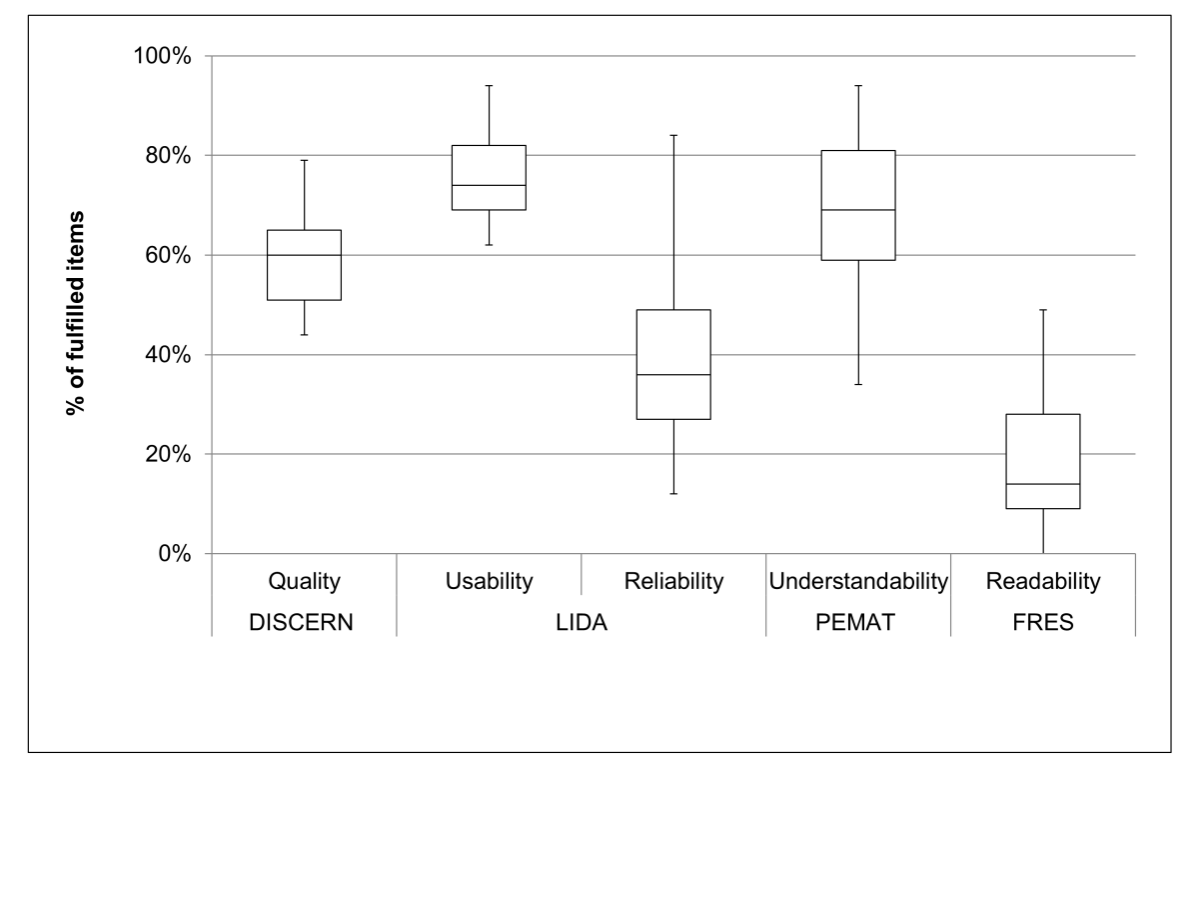
Quality, usability, reliability, understandability, and readability of 45 websites providing information on melanoma immunotherapy. Box, lower line: quartile Q1 (25% quantile), middle line: median, upper line: Q3 (75% quantile); aerials, highest values. PEMAT: Patient Education Materials Assessment Tool; FRES: Flesch Reading Ease Score.

The 30 domains scored between 39 and 67 points (maximum of 81 points possible). The assessment by LIDA and the separate analysis of the usability and reliability sections indicated that the usability criteria (74%; mean (SD): 40 (2.0) out of 54 points) were more frequently fulfilled than the reliability criteria (38%; 10 (1.6) out of 27 points; Multimedia Appendix 4; Figure 1). In particular, the currency and conflicts of interest criteria were least met (Multimedia Appendix 5). Medecon.ruhr and scinexx.de were the domains that received the lowest overall LIDA scores (39 points each), aerzteblatt.de and krebsgesellschaft.de were rated highest (67 and 66 points; Multimedia Appendix 2; #22, #18, #1, and #2).

### Understandability and Actionability—PEMAT Results

On average, 69% of the understandability elements were applied by the 45 websites. The lowest PEMAT score was received from journalonko.de (34%) and the highest from uniklinik-ulm.de (94%; Multimedia Appendix 2; #9 and #26). The reviewers could assess an item of actionability in only 16% (7/45) of websites, indicating that actionability was nonexistent in nearly all identified websites.

### Readability—Flesch Reading Ease Scores

The median FRES was 14 (IQR: 8.5-28.0), indicating that the information of at least 50% (23/45) of the 45 websites was very difficult to read for laypersons. Receiving a FRES of 49, the most readable text was provided by swr.de, whereas the lowest FRES was calculated for the text provided by a link from krebsgesellschaft.de with a score of –15 (Multimedia Appendix 2; #6 and #2).

### Interrater Agreement

We determined intraclass correlation coefficients of .831 to .964, indicating a high overall interrater agreement concerning the assessment by DISCERN, LIDA, and PEMAT (Multimedia Appendix 6) [35]. The interitem correlations *r* varied between.401 and.974, indicating moderate to a high individual agreement among the 3 reviewers when assessing the individual items.

### Popularity and Visibility

The majority of users visited the domains almost twice a day [median, 1.7 (IQR: 1.6-2.2)] and browsed a website between 22 and 350 seconds and 142 seconds on average. The domain t-online was the most frequently and longest visited of the domains considered and was ranked the most popular according to the ALEXA tool. The least popular website considered was journalonko.de (Multimedia Appendix 2; #9). Wikipedia.de showed the highest SISTRIX visibility value (Multimedia Appendix 2; #8). Multimedia Appendix 4 summarizes the results of the assessments using DISCERN, PEMAT, and LIDA by 3 independent reviewers and the determined FRES, ALEXA (Germany), and SISTRIX values.

### Subgroup Analyses

#### Differences Between Domains of Different Providers

In addition to significant differences in popularity, visibility (*P*=.02), and daily visit values (*P*=.02), differences between the websites of different providers were particularly evident from the readability (*P*<.001) and understandability scores (*P*<.001), indicating that the links of the noncommercial provider had the lowest readability and general public press the highest readability (Multimedia Appendix 7). However, pharmaceutical companies, hospital or health professional, and Wikipedia were most keen in applying understandability elements. DISCERN (between 49 (SD 7) and 62 (SD 1) points; *P*=.07) and LIDA (between 46 (SD 4) and 66 points; *P*=.12) scores were not significantly different.

#### Differences Between General Public Domains and Oncology Expert Domains

Websites addressing the general public had significantly higher popularity (ALEXA; *P*<.001) and visibility (SISTRIX) ranks (*P*=.001) and were visited longer on average (*P*=.04) but not necessarily more often (*P*=.23) than websites predominantly addressing or provided by oncology experts. The most visible and popular oncology expert domains were pharmazeutische-zeitung.de, aerzteblatt.de, and krebsgesellschaft.de. Furthermore, the public domains had better readability (*P*<.001) and understandability (*P*=.002; Multimedia Appendix 8). Significant differences in terms of quality, usability, and reliability could not be detected (*P*=.06-.76). However, the LIDA scores were marginally in favor of oncology expert domains.

## Discussion

### Principal Findings and Comparison With Prior Work

The websites that we have systematically identified provided information on melanoma immunotherapy as the main subject or reported on aspects of cancer immunotherapy in general. Nearly half of the identified websites were published in 2017, and the other half in the years before. The majority of websites could be assigned to providers of scientific medical information or the general public press and Wikipedia.

We assessed the quality of the individual website information as medium–low to medium–high, and we found only 2 website domains from the health care sector that displayed a quality certification. These findings are similar to those of Bari et al [19], who found a low use of quality certificates on German websites (in 4 out of 21), providing general Web-based information on melanoma. An explanation for this persisting low presence of certified websites might be that webmasters have to register, apply, and—following the acceptance—pay for certification. For example, to acquire the HONcode certificate, webmasters recurrently have to apply for certification. In addition, from the second HONcode membership, a fee is due [36]. Hence, the awarding process of such quality certificates proclaiming trustworthiness should be kept in mind, and either their presence or their absence should be critically appraised. However, the 2 domains we found providing quality certificates received the above-average quality of information, usability and reliability scores, and thereby can be seen as a kind of certificate affirmation.

Score deductions resulting in an overall mediocre quality of the websites’ content mainly resulted from incomplete and sometimes superficial reporting about melanoma immunotherapy, characterized by missing information on possible treatment consequences for the patients’ quality of life, on the consequences of nontreatment, and on unclear scientific evidence for different aspects of treatment. Conversely, more effort was made to describe the effects and benefits of treatment; this is a fairly known problem with Web-based cancer information [20-22,37-39], which makes reporting one-sided and, thus, withholds important information for treatment decision making. However, this may not apply to all websites considered. Highly variable content and quality of websites providing general melanoma information have been reported recently [40].

In terms of content, websites offered to the general public provided only some aspects of melanoma immunotherapy, whereas websites offered by and to oncology experts included more detailed and substantial information. In addition, oncology expert domains marginally met more reliability and usability criteria. Overall, the 30 domains demonstrated high usability but low reliability and even lower readability. The low readability may be attributed to a frequent application of medical terms, which are not explained in layperson’s terms. In addition, sentences were sometimes very long and nested, especially in oncology expert websites, which makes the readability more complicated. This pattern of high usability but low reliability and readability has also been found to be typical for websites providing cancer information [20,21,24,39], including those providing information on melanoma [16,19]. It is fundamental that patients can easily read treatment text and understand the medical terms to benefit from the information. Furthermore, an indication of the sources quoted and their recency should routinely be provided on a website to enhance reliability and trustworthiness. Another problem that should also be addressed in this context is that among the websites published before 2017, there were websites offering information dating back to 2007. As immunotherapy is such a novel innovation and because a lot of progress has been evolved since the approval for melanoma treatment with immune checkpoint-inhibiting agents, such as ipilimumab in 2011, the content of such old websites is questionable and unreliable.

In general, and if appropriate, the identified websites showed good efforts to make their contents understandable by applying various visual aids and supportive structuring elements (eg, illustrations, paragraphs, simple numbers, and short sections of texts). However, we found very few elements of actionability. In this regard, the most visible German websites on melanoma immunotherapy were in line with other patient education materials that were previously assessed by the same tool [41,42]. However, the application of more elements of actionability and interactivity (eg, checklists, videos, and webinars) may facilitate the users’ handling and understanding of difficult website content and is highly recommended.

Overall, we found that websites that addressed the general public were superior in terms of the popularity and visibility compared with oncology expert domains. They applied more elements to support the understandability and their information provided on immunotherapy was easier to read for laypersons but more superficial in terms of the content. However, we found no discrepancy between the visibility and quality of websites, as this was the case in a previous study on German Web-based cancer information [39].

### Strengths

To date, no work has been published with a comprehensive and thorough assessment of the most visible German websites providing information on melanoma immunotherapy. Our results updated the currently available evidence on the Web-based melanoma information quality. Three reviewers have independently assessed the websites using a variety of validated instruments, yielding an overall good interrater agreement. We have not only assessed the websites subjectively (eg, by using tools like DISCERN, PEMAT, or LIDA) but also evaluated them objectively by using tools like FRES, SISTRIX, or ALEXA, which measure the values quantitatively. As immunotherapy is such a novel treatment approach and given its complexity, a comprehensive assessment of the most visible websites addressing this topic is of high relevance for both patients and physicians.

Furthermore, 2 researchers have searched the search engines Google, Bing, and Yahoo because these websites are searched from the most patients and the public for general cancer information [12-15]. In addition, it is interesting to note that the 2 researchers were at different geographical locations of Germany when searching the 3 search engines. Hence, we might have identified a full overview on websites because of different GPS data affecting the algorithms of the search engines. Therefore, our identified websites might be more comprehensive.

### Limitations

We are aware that this study has some limitations. First, the websites identified represent only a snapshot of the time when we searched the 3 search engines in July 2017. Bearing in mind that immunotherapy is a quite novel approach with a lot of ongoing progress involved and considering the fast-moving nature of the internet, one might not identify the identical websites as we identified. However, we are confident that the majority of websites will still be available at a later time. Second, we might have either overestimated or underestimated FRES values, as we only extracted a sample of 200-500 words (or 4-5 sentences) to determine the score. It may be possible that the calculated value might differ when using the entire number of words available on the website. However, regarding the consistency, we have stuck to our predefined number of words. Third, we did not include websites of restricted access (eg, asking for log-in). Therefore, we might have failed to identify further websites that were only available when having access. Hence, the list of identified websites might be incomplete. However, we believe that patients with melanoma would not consider those websites and would rather acquire information from easily-accessible websites. Finally, the DISCERN, LIDA, and PEMAT assessment was a result of subjectivity introduced by the individual perspectives of the 3 reviewers. However, the high interrater agreement suggests that most of the independently detected deficits were apparent to all of the reviewers and, thus, may be problematic for others.

### Conclusions

In general, German websites on immunotherapy for patients with melanoma provide inexpensive and easily accessible means to acquire disease- and treatment-specific information. We found the most visible among them to be user-friendly and understandably structured. However, the optimization of the most visible websites is desirable; in particular, improvement of the information readability and more provision of meta-information to increase the reliability. We suggest that the ideal websites should be a hybrid and should include both oncology expert parts for completeness, content-related integrity, and details, as well as general public press parts for the visibility and comprehensibility to be beneficial for more patients.

## Appendix

Multimedia Appendix 1 Flowchart of the website identification process according to the PRISMA guidelines.

Multimedia Appendix 2 The summary of information about German websites on melanoma immunotherapy analyzed in this study.

Multimedia Appendix 3 Summary of the individual DISCERN item assessment of the most visible German websites (n = 45) on melanoma immunotherapy.

Multimedia Appendix 4 The assessment summary of the most accessible German websites on melanoma immunotherapy.

Multimedia Appendix 5 Results of LIDA assessment on usability and reliability of German website domains (n = 30) on melanoma immunotherapy.

Multimedia Appendix 6 Interrater agreement.

Multimedia Appendix 7 Readability (Flesch Reading Ease scores) differences between the 45 websites.

Multimedia Appendix 8 Differences between oncology expert domains and general public domains.

## Abbreviations

FRES: Flesch Reading Ease Score
IQR: interquartile range
NMSC: nonmelanoma skin cancer
PEMAT: Patient Education Material Assessment Tool
