# Supplementary figures and images for: Exploring the Most Visible German Websites on Melanoma Immunotherapy: A Web-Based Analysis
Source: JMIR Cancer. 2018 Dec 13;4(2):e10676. doi: 10.2196/10676 (PMC6315239; doi:10.2196/10676)

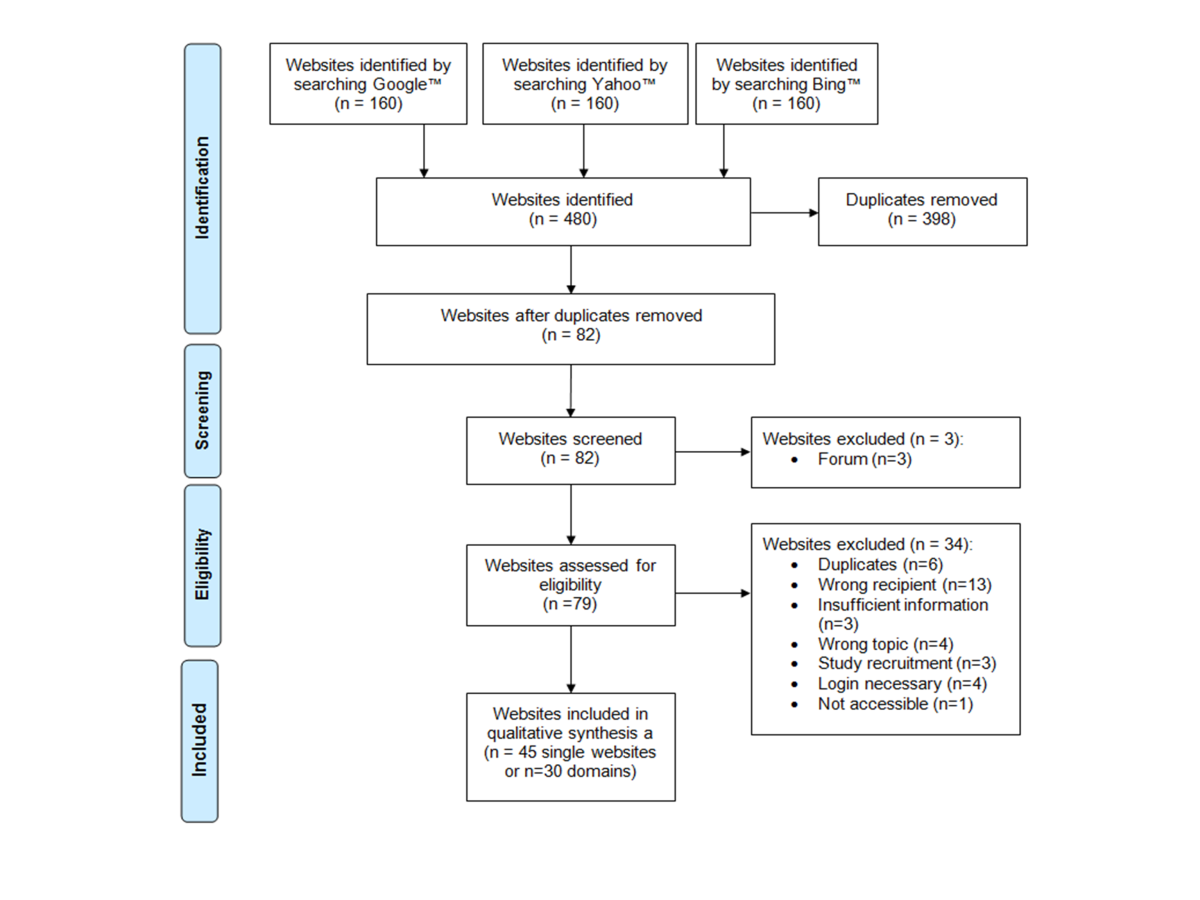

Supplement: Multimedia Appendix 1 [file cancer_v4i2e10676_app1.png]

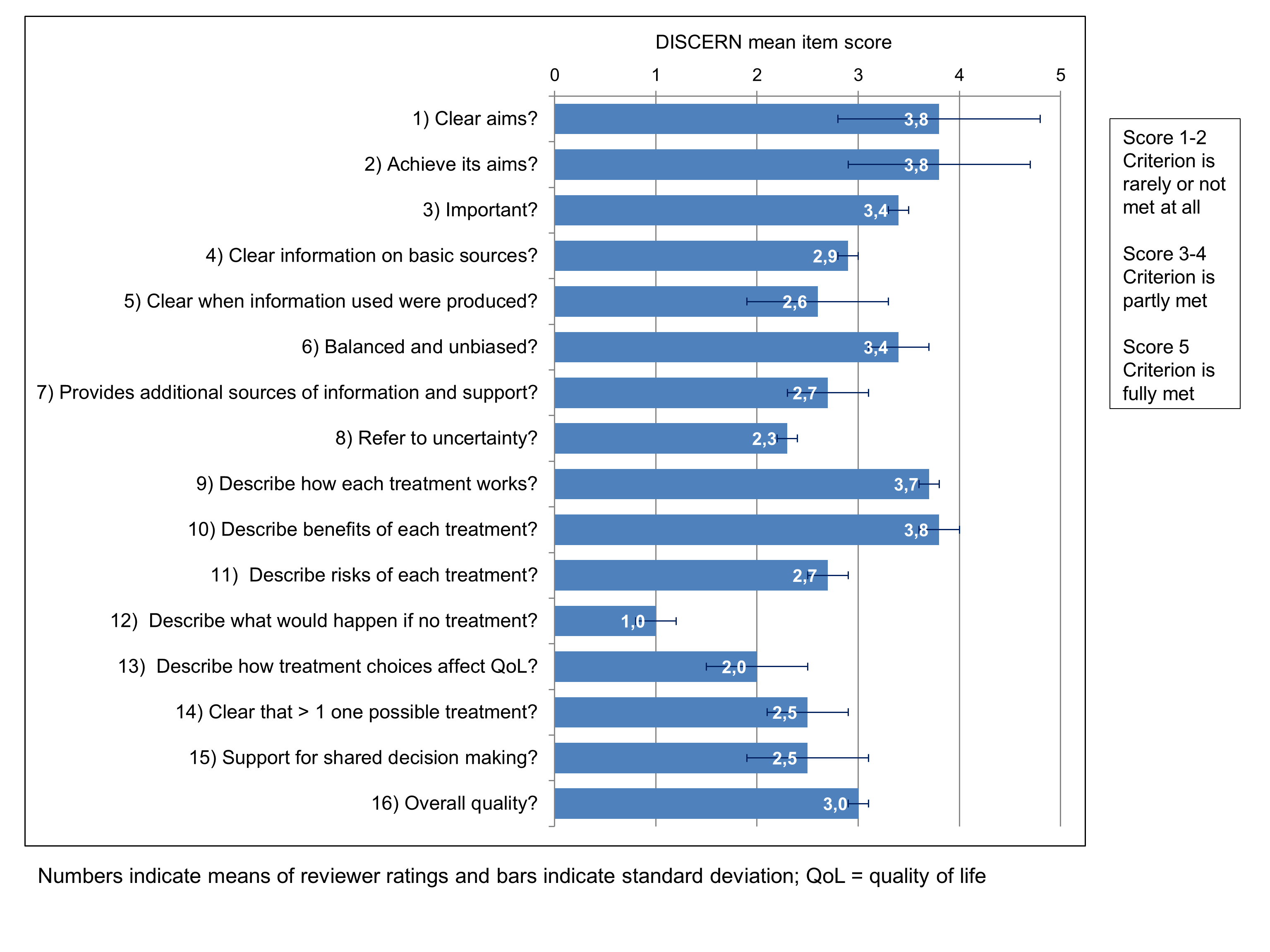

Supplement: Multimedia Appendix 3 [file cancer_v4i2e10676_app3.png]

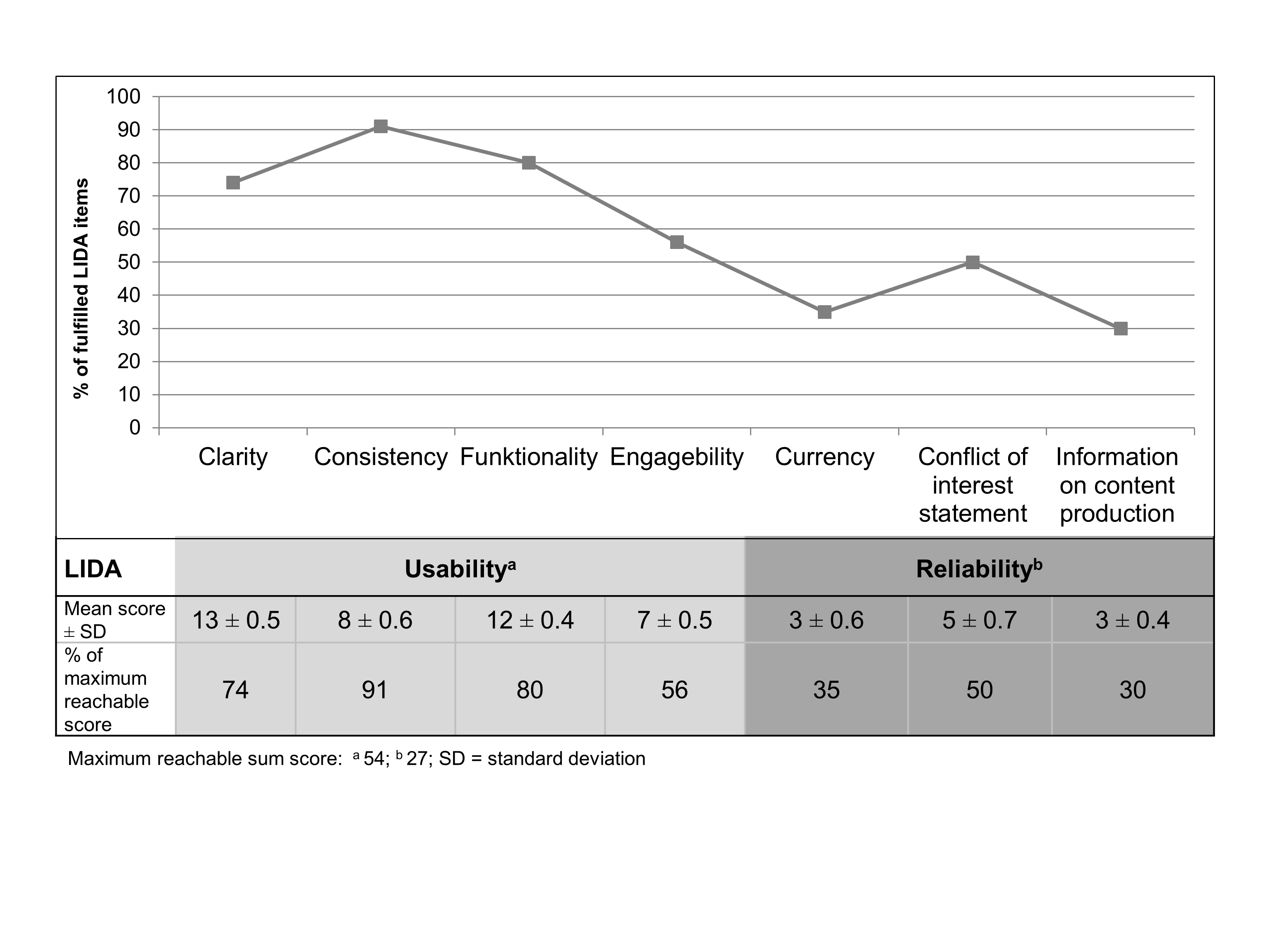

Supplement: Multimedia Appendix 5 [file cancer_v4i2e10676_app5.png]

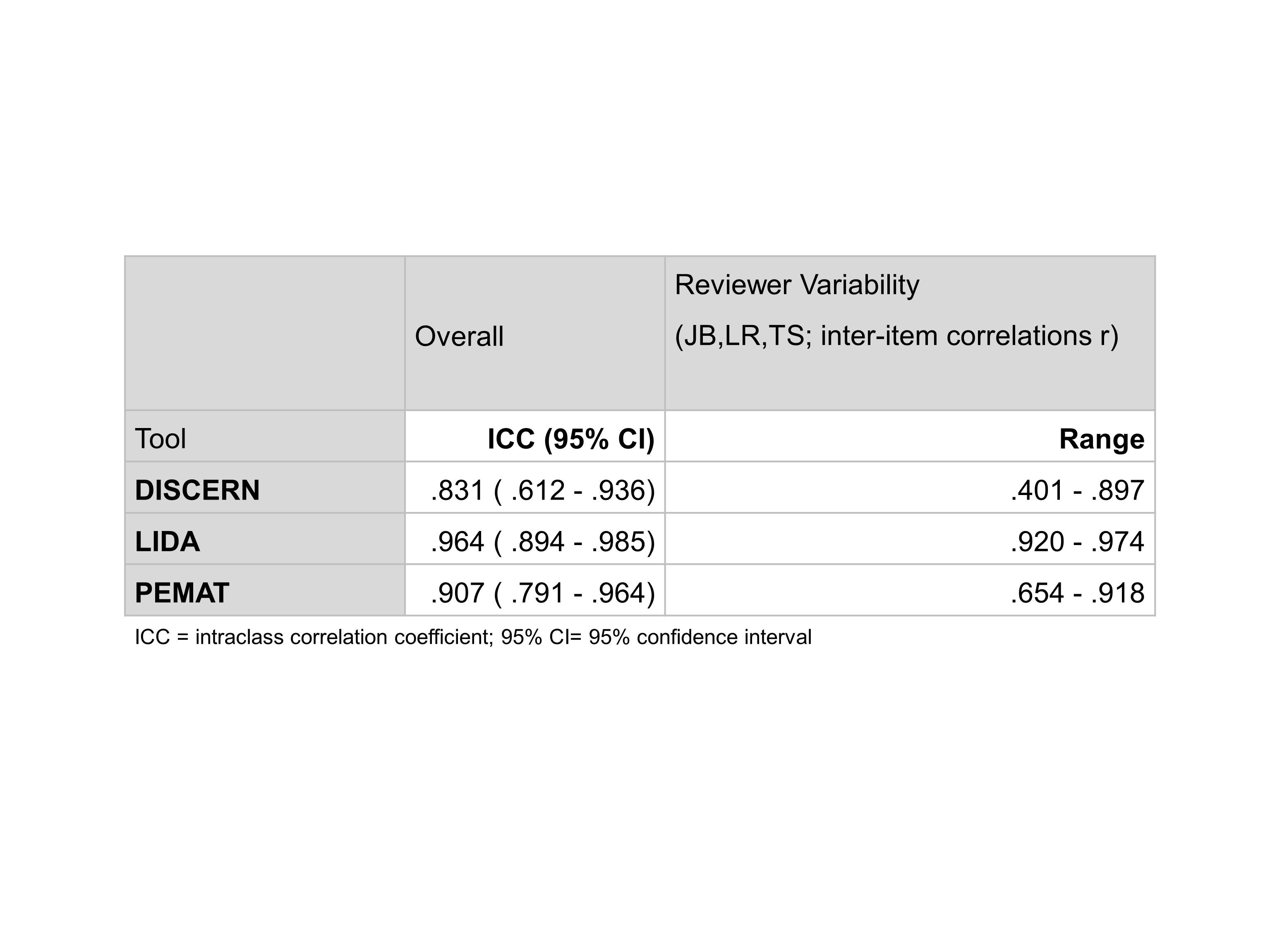

Supplement: Multimedia Appendix 6 [file cancer_v4i2e10676_app6.png]

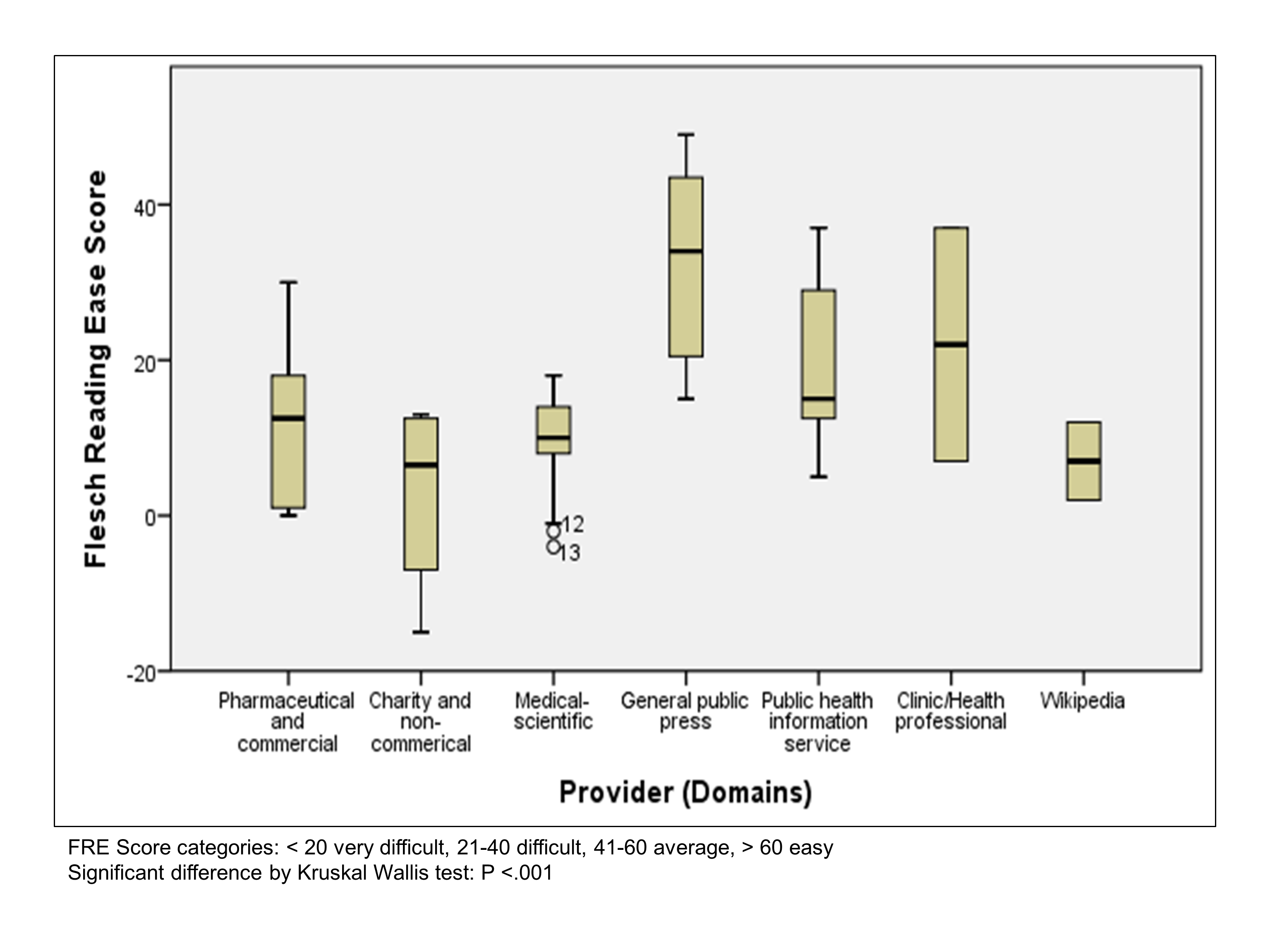

Supplement: Multimedia Appendix 7 [file cancer_v4i2e10676_app7.png]

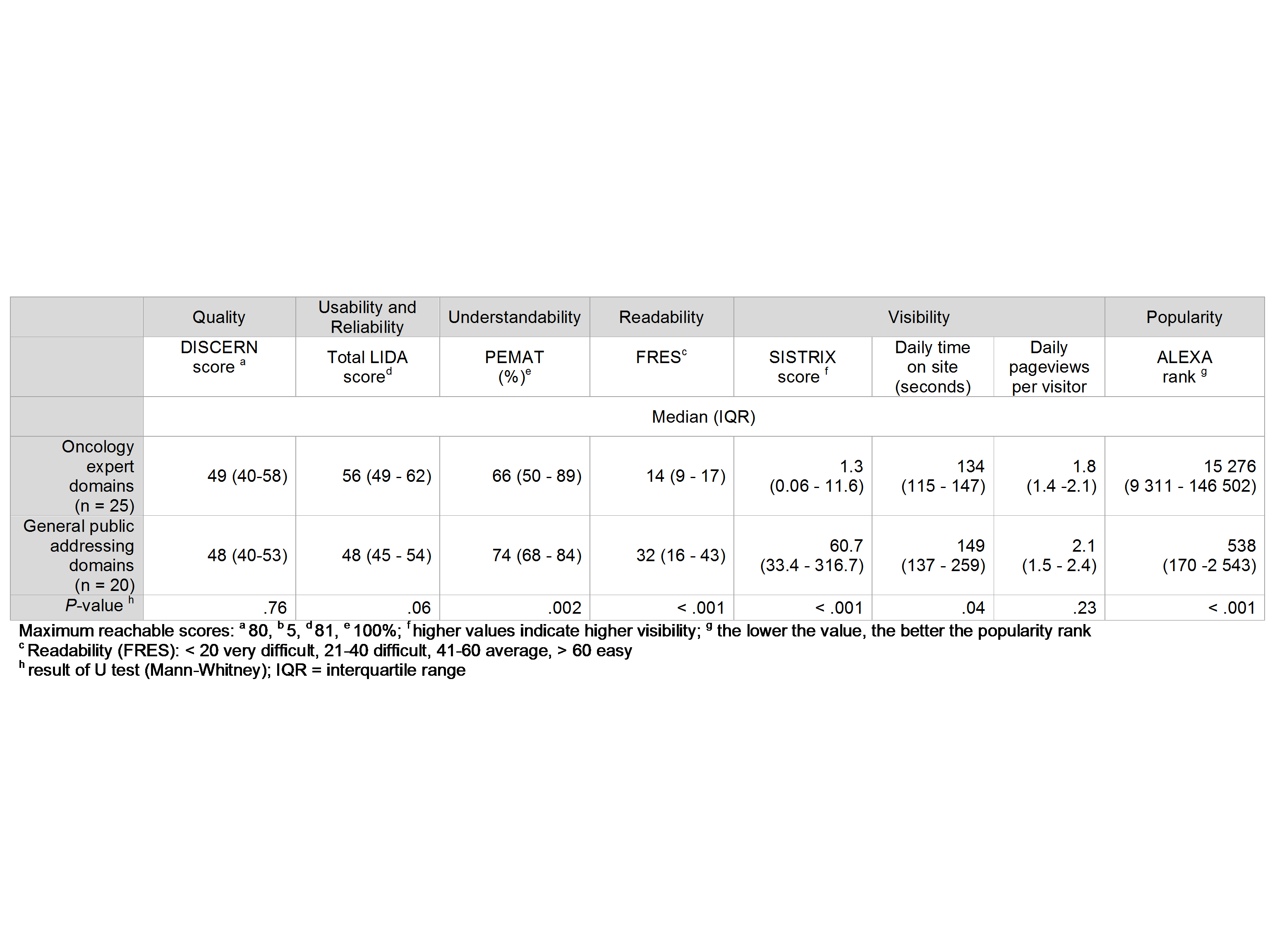

Supplement: Multimedia Appendix 8 [file cancer_v4i2e10676_app8.png]
