# Supplementary material for: Exploring the Most Visible German Websites on Melanoma Immunotherapy: A Web-Based Analysis
Source: JMIR Cancer. 2018 Dec 13;4(2):e10676. doi: 10.2196/10676 (PMC6315239; doi:10.2196/10676)
Supplement: Multimedia Appendix 2 [file cancer_v4i2e10676_app2.pdf]

Nr	Domain	Provider category	Individual website (URL)	Year created	Quality	Validity (Usability and Reliability)	Readability	Popularity rank	Visibility rank
					(DISCERN Score)[a]	LIDA Score[b]	(FRES)[c]	(ALEXA Score Germany)[d]	(SISTRIX Score)[e]
1	aerzteblatt.de	3	aerzteblatt.de/archiv/161328/Immuntherapie-des-Metastasierten-Melanoms-Hohe-Antitumorwirkung-belegt (Archived at http://www.webcitation.org/6y3npwT8R)	2017	48	67	8	2 268	14.10
			aerzteblatt.de/archiv/168542/Malignes-Melanom-Kombinations-Immuntherapie-ist-hochwirksam (Archived at http://www.webcitation.org/6y3nrdeqQ)	2017	43		18		
			aerzteblatt.de/archiv/181778/Immuntherapie-bei-metastasiertem-malignen-Melanom-Ein-Drittel-spricht-auf-PD-1-Inhibitor-trotz-Vortherapie-an (Archived at http://www.webcitation.org/6y3ntBaTz)	2017	43		9		

			aerzteblatt.de/nachrichten/45262/USA-Lebensverlaengernde-Immuntherapie-bei-Melanom-zugelassen (Archived at http://www.webcitation.org/6y3oFKfEM)	2017	50		10		
			aerzteblatt.de/nachrichten/54640/Melanom-Neue-Immuntherapien-versprechen-schnelle-Remissionen (Archived at http://www.webcitation.org/6y3oGW9NO)	2017	51		14		
			aerzteblatt.de/nachrichten/58919/Melanom-Immuntherapien-zeigen-langfristige-Wirkungen (Archived at http://www.webcitation.org/6y3oI20YU)	2017	53		13		
2	krebsgesellschaft.de	2	krebsgesellschaft.de/onko-internetportal/aktuelle-themen/news/immuntherapie-beim-malignen-melanom.html (Archived at http://www.webcitation.org/6y3pGSa2B)	2014	49	66	12	15 276	11.11
			krebsgesellschaft.de/onko-internetportal/aktuelle-themen/news/melanom-immuntherapie-	2014	49		-15		

			kombiniert-noch-wirksamer.html (Archived at http://www.webcitation.org/6y3pj3wL0)						
			krebsgesellschaft.de/onko-internetportal/aktuelle-themen/news/schwarzer-hautkrebs-adjuvante-immuntherapie-bei-lymphknotenbefall.html (Archived at http://krebsgesellschaft.de/onko-internetportal/aktuelle-themen/news/schwarzer-hautkrebs-adjuvante-immuntherapie-bei-lymphknotenbefall.html)	2014	49		1		
			krebsgesellschaft.de/onko-internetportal/aktuelle-themen/news/schwarzer-hautkrebs-nebenwirkungen-bei-immuntherapie.html (Archived at http://www.webcitation.org/6y3qGJtQd)	2014	50		13		
3	immuntherapiekrebs.de	3	immuntherapiekrebs.de/immuntherapie-hautkrebs/ (Archived by WebCite® at http://www.webcitation.org/6y3qX5z8V)	2016	39	50	12	no data available	0.00
			immuntherapiekrebs.de/	2016	40		-4		

			mehr-lebensqualitaet-mit-immuntherapie-bei-malignem-melanom/ (Archived at http://www.webcitation.org/6y3rHVNdK)						
			immuntherapiekrebs.de/schwarzer-hautkrebs-kombination-von-strahlentherapie-und-immuntherapie/ (Archived at http://www.webcitation.org/6y3rWnlL4)	2016	45		-2		
4	onkologie.hexal.de	1	onkologie.hexal.de/arzneimittel/immuntherapie/ (Archived at http://www.webcitation.org/6y3rl6nRg)	2017	38	44	15	14 685	8.83
			onkologie.hexal.de/arzneimittel/immuntherapie/nebenwirkungen.php (Archived at http://www.webcitation.org/6y3sSWfVS)	2017	47		10		
5	apotheken-umschau.de	5	apotheken-umschau.de/Krebs/Immuntherapie-gegen-Krebs-532029.html (Archived at http://www.webcitation.org/6y3tRclss)	2017	61	63	37	537	220.30
			apotheken-umschau.de/Melanom/Malignes-Melanom-	2013	50		5		

			Therapie-49584_5.html (Archived at http://www.webcitation.org/6y6oQWQVr)						
6	swr.de	4	swr.de/odysso/immuntherapie/-/id=1046894/did=14757824/nid=1046894/1aov4nj/index.html (Archived at http://www.webcitation.org/6y6oXWVYM)	2015	55	44	49	504	55.37
			swr.de/landesschau-rp/gut-zu-wissen/spritze-statt-skalpell-neue-immuntherapie-gegen-hautkrebs/-/id=233210/did=19060098/nid=233210/1ox3qnh/index.html (Archived at http://www.webcitation.org/6y6pA0koL)	2017	51		27		
7	welt.de	4	welt.de/gesundheit/article132175869/Neue-Therapien-gegen-toedlichen-schwarzen-Hautkrebs.html (Archived at http://www.webcitation.org/6y6pVnBIT)	2014	38	48	34	39	347.91
			welt.de/gesundheit/article139170518/Melanom-laesst-sich-mit-einer-Impfung-baendigen.html (Archived at http://www.webcitation.or	2015	39		15		

			g/6y6qDbD7u)						
8	wikipedia.org	7	wikipedia.org/wiki/Krebsimmuntherapie (Archived at http://www.webcitation.org/6yOq2xh8I	2017	63	58	12	1 314	6871.92
			wikipedia.org/wiki/Malignes_Melanom (Archived at http://www.webcitation.org/6y6qewIbN)	2017	61		2		
9	journalonko.de	3	journalonko.de/news/anzeigen/10642 (Archived at http://www.webcitation.org/6y6qjiQNT)	2017	39	56	10	192 675	0.03
10	keyopinions.info	3	keyopinions.info/downloads/malignes-melanom/ (Archived at http://www.webcitation.org/6y6rh56xp)	no data available	48	47	-1	no data available	0.00
11	pharmazeutische-zeitung.de	3	pharmazeutische-zeitung.de/index.php?id=2451 (Archived at http://www.webcitation.org/6y6spjwC1)	2007	41	45	14	5 464	12.12
12	rosenfluh.ch	3	rosenfluh.ch/media/dermatologie-aesthetische-medizin/2015/03/Immuntherapie_beim_malignen_Melanom.pdf (Archived at http://www.webcitation.org/6y6tiZ0kY)	2015	58	53	14	130 997	0.07

13	augsburger-allgemeine.de	4	augsburger-allgemeine.de/wissenschaft/Immuntherapie-gegen-Krebs-hat-deutliche-Nebenwirkungen-id40285822.html (Archived at http://www.webcitation.org/6y6trvo6B)	2017	44	45	16	539	45.68
14	br.de	4	br.de/br-fernsehen/sendungen/gesundheit/krebstherapie-immuntherapie-krebs-fortschritt-100.html (Archived at http://www.webcitation.org/6y6uRQnBu)	2016	51	46	44	327	69.69
15	daserste.de	4	daserste.de/information/wissen-kultur/w-wie-wissen/sendung/krebs-immuntherapie-100.html (Archived at http://www.webcitation.org/6y6uUkYba)	2015	44	50	46	214	37.15
16	deutschlandfunk.de	4	deutschlandfunk.de/die-scharfmacher-immuntherapien-gegen-krebs.740.de.html?dram:article_id=352851	2016	52	53	43	831	22.01
17	focus.de	4	focus.de/gesundheit/ratgeber/krebs/therapie/immuntherapie-schwere-nebenwirkungen-der-	2015	50	50	15	36	422.2

			immuntherapie_id_4573252.html (Archived at http://www.webcitation.org/6y6vDybvD)						
18	scinexx.de	4	scinexx.de/wissen-aktuell-21629-2017-07-06.html (Archived at http://www.webcitation.org/6yCbPYm9K	2017	41	39	25	8 343	1.48
19	t-online.de	4	t-online.de/gesundheit/krankheiten-symptome/krebs/id_72666092/krebs-immuntherapie-wirkt-bei-schwarzem-hautkrebs.html (Archived at http://www.webcitation.org/6yCbafmNC)	2015	37	43	38	17	306.36
20	gesundheitsstadt-berlin.de	5	gesundheitsstadt-berlin.de/immuntherapie-bei-melanom-kombination-wirkt-besser-6523/ (Archived at http://www.webcitation.org/6yCblKvlG)	2015	46	57	15	32 403	2.04
21	malignes-melanom.org	5	malignes-melanom.org/therapie/ (Archived at http://www.webcitation.org/6yCc3ZurL)	2017	36	49	14	no data available	0.00

22	medecon.ruhr	5	medecon.ruhr/2017/07/immuntherapie-bei-schwarzem-hautkrebs/ (Archived at http://www.webcitation.org/6yCcOGzMN)	2017	35	39	11	no data available	0.00
23	planet-wissen.de	5	planet-wissen.de/gesellschaft/krankheiten/krebs/immuntherapie-krebs-patient-100.html (Archived at http://www.webcitation.org/6yCcUcgmg)	2017	57	45	29	2 575	65.94
24	praxisvita.de	5	praxisvita.de/immuntherapie-gegen-hautkrebs-impfung-mit-erfolg (Archived at http://www.webcitation.org/6yCcdUdcA)	2017	35	47	29	2 532	41.03
25	krebsmagazin.de	6	krebsmagazin.de/immuntherapie-in-der-onkologie-neue-standards-und-zukunftige-strategien/ (Archived at http://www.webcitation.org/6yCcpcVhZ)	2015	47	48	7	no data available	0.01
26	uniklinik-ulm.de	6	uniklinik-ulm.de/fileadmin/Kliniken/Dermatologie/Texte/HTZ/Krebsliga_Schweiz_Melanomflyer.pdf (Archived at http://www.webcitation.org/6yCdJM3VI)	2008	63	57	37	13158	1.04

27	roche.de	1	roche.de/pharma/onkologie/hautkrebs/therapie/schwarzer-hautkrebs/immuntherapie.html (Archived at http://www.webcitation.org/6yCdOKWae)	2017	54	55	18	33 307	1.25
28	aimatmelanoma.org	1	aimatmelanoma.net/de/aim-for-answers/behandlung-des-melanoms/pruefpraeparate/immuntherapie.html (Archived at http://www.webcitation.org/6yCdY205f)	2016	52	58	0	no data available	0.00
29	krebs.de	1	krebs.de/malignes-melanom/therapie (Archived at http://www.webcitation.org/6yCdaLlnl)	no data available	58	56	1	162 007	0.05
30	wissen-immuntherapie.de	1	Wissen-immuntherapie.de/einsatzgebiete/hautkrebs/ (Archived at http://www.webcitation.org/6yCdpwNqH)	2016	46	50	30	no data available	0.00

Provider categories: 1, commercial and pharmaceutical companies; 2, noncommercial/charity; 3, medical or scientific; 4, general public press; 5, commercial health information services; 6,clinics or health professionals; 7, Wikipedia.

[a] The higher the value, the better the quality (maximum of 80 points).

[b] Maximum reachable LIDA score: 81.

[c] The higher the value, the easier to read (maximum of 100 points).

[d] The lower the value, the better the popularity rank.

[e] Higher values indicate higher visibility.
