# Supplementary material for: Exploring the Most Visible German Websites on Melanoma Immunotherapy: A Web-Based Analysis
Source: JMIR Cancer. 2018 Dec 13;4(2):e10676. doi: 10.2196/10676 (PMC6315239; doi:10.2196/10676)
Supplement: Multimedia Appendix 4 [file cancer_v4i2e10676_app4.pdf]

Category	Quality		Usability	Reliability	Understandability	Readability	Popularity	Visibility		
Tool	DISCERN Score[a]	Mean DISCERN Score[b]	LIDA Score[c,d]		PEMAT (%)[e]	FRES[f]	ALEXA Rank[g]	SISTRIX Score[h]	Daily time on site (seconds)	Daily pageviews per visitor
n	45	45	30	30	45	45	23	24	23	29
Mean (SD)	48 (7.6)	2.8 (0.4)	40 (2.0)	10 (1.6)	69 (16)	17 (14)	26,959 (55,069)	285 (1228)	158 (69)	1.9 (0.1)
Median (IQR)	48 (41-52)	2.8 (2.4-3.1)	41 (37-42)	11 (8-12)	69 (58.5-80.5)	14 (8.5-28)	2532 (504-15,276)	10 (0-58)	141.5 (127.5-160.5)	1.7 (1.6-2.2)
Min	35	2.1	28	1	34	-15	17	0	22	1.0
Max	63	3.7	54	24	94	49	192,675	6872	350	3.4
Category	mediocre		good	low	good	very low				

SD = standard deviation, IQR, interquartile range; Min, minimum of ratings; Max, maximum of ratings.
Maximum reachable scores: [a]80, [b]5, [c]54, [d]27, [e]100%.
[h]Higher values indicate higher visibility.
[g]The lower the value, the better the popularity rank.
[f]Readability (FRES): <20 very difficult, 21-40 difficult, 41-60 average, >60 easy.

Multimedia Appendix 4. The assessment summary of the most accessible German websites on melanoma immunotherapy.
